# Managing the urinary tract in spinal cord injury

**DOI:** 10.4103/0970-1591.65399

**Published:** 2010

**Authors:** Simon C. W. Harrison

**Affiliations:** Department of Urology, Pinderfields Hospital, Wakefield, West Yorkshire, UK

**Keywords:** Neurogenic urinary bladder, spinal cord injury, urinary tract, urinary bladder, urodynamics, urinary calculi, urinary tract infection

## Abstract

This review sets out to provide an overview of the author’s approach to the management of the urinary tract in the patient who has suffered from an injury to their spinal cord. Emphasis is given to the need to understand the fundamental pathophysiological patterns that are seen with injuries that involve the sacral segments of the cord (the conus) and those that spare the conus but interrupt communication between the sacral parasympathetic and somatic centers and the brain (supraconal lesions). The importance of patient participation in management decisions is highlighted by considering the different ways in which the urinary tract can be managed and how the clinician needs to try to meet patient expectations and requirements while establishing safe urological management. Finally, consideration is given to the importance of establishing an appropriate follow up regime and managing urinary tract complications effectively.

## INTRODUCTION

The survival chances of patients who suffer from an injury to their spinal cord have dramatically improved over the last seven decades and there is no doubt that improved urinary tract management has played a significant role in this process.[1,2] General advances have included the development of antibiotics, the introduction of better materials and designs for catheters and appliances and the adoption of effective upper urinary tract surveillance with urography and now, ultrasonography. However, a key specific change has been the introduction of urodynamic assessment of lower urinary tract function and the subsequent understanding of the pathophysiology and patterns of lower urinary tract dysfunction that accompany spinal cord injury (SCI).[3,4]

It should also be appreciated that the passage of time has seen changes in the approach that clinicians have adopted when addressing the problem of how to manage the urinary tract in SCI. Spinal cord injury centers were first developed in an era when doctors’ opinions were rarely challenged. The early dramatic improvement in outcomes was cemented in place by the adoption of a somewhat dogmatic approach to urinary tract management. These rigid attitudes are now being replaced by a greater degree of flexibility so that clinician and patient can work in a partnership that should produce a urinary tract management system that is acceptable to the patient and minimizes the long-term risk of dangerous complications.

On a global scale, spinal cord injury remains a condition with outcomes that vary widely; the prognosis for patients in undeveloped healthcare systems remains disastrous.[5] There is clearly a need to adapt care systems for SCI patients from the centers of excellence for environments where healthcare resources are limited.

Finally, it must be acknowledged that modern urinary tract management in SCI is far from perfect. Significant rates of complications such as urinary tract infection (UTI) and stones persist.[6,7] Further, the evidence base for many aspects of urological care in SCI is weak with few relevant, high-quality clinical trials having been conducted.

## PATHOPHYSIOLOGY

It is convenient to consider the pathophysiology of the lower urinary tract after SCI in relation to the initial phase after injury, when spinal shock is seen, and then with respect to the typical functional patterns that are seen in patients with complete injuries to the spinal cord. Finally the effect of incomplete SCI can be considered. The work of the International Continence Society Standardization Committee has been of enormous importance as they have provided clarity in relation to the definition of the nomenclature surrounding lower urinary tract dysfunction.[8]

Spinal shock is defined as the sudden cessation of spinal reflex activity in areas below the level of injury. The effect of spinal shock is to render the lower urinary tract areflexic. The bladder will fill passively until overflow incontinence occurs. The duration of this phase is variable so that in some patients with supraconal SCI, bladder reflex activity may emerge within days while in others, many weeks can pass before reflex function is seen.

Complete injuries that ablate the conus (sacral segments) of the spinal cord or destroy the cauda equina are typically seen in patients with injuries involving the lumbar region of the spine bearing in mind that the tip of the spinal cord is found at the level of the L1/L2 vertebrae. However, injuries at higher levels can also lead to loss of sacral reflex function through damage to the blood supply to the distal spinal cord. Patients with conus injuries will have a loss of sacral reflex activity which will typically produce a flaccid paraplegia, reduced anal tone and loss of sensation in the sacral dermatomes.

Video-urodynamic evaluation in the patient with a complete conus injury will usually demonstrate the following features:

- No reflex bladder activity during filling
- Compliance that is usually normal but is reduced in a significant minority of patients
- Absent or greatly reduced awareness of filling
- An open bladder neck with stress incontinence is seen in most patients. Continence is dependant on residual tone in the smooth muscle component of the distal sphincter mechanism; a high level of tone can be termed non-relaxing urethral sphincter obstruction.
- Voiding either does not occur or is achieved by abdominal straining.

In contrast, a patient with a complete SCI at a level above the conus and in whom the conus remains viable, will have a distal segment of spinal cord that is disconnected from higher neurological centers but which continues to function independently from those centers – the distal autonomous cord. The disconnection between the parasympathetic and somatic centers in the sacral cord from the centers in the pons and peri-aquaductal gray matter in the brain leads to a lack of coordination between detrusor contraction and sphincter relaxation during voiding. Clinical examination will reveal evidence of a spastic paraplegia or quadriplegia with normal rectal tone and absent sensation below the injury level.

The results of video-urodynamic assessment will typically show:

- Involuntary, reflex detrusor contractions during filling (neurogenic detrusor overactivity) with accompanying reflex contraction of the striated distal sphincter (detrusor sphincter dyssynergia - DSD). On X-ray screening in men, the bladder neck and prostatic urethra will open during detrusor contractions but the distal sphincter will remain closed at first but may subsequently relax and allow urine to be voided. The degree of DSD varies between patients so that some patients will have a complete absence of voiding during detrusor contractions while others will have minimal DSD with efficient bladder emptying being seen.
- Bladder compliance is usually normal.
- Absent or greatly reduced awareness of filling.
- Voiding by involuntary reflex contractions as above or by stimulated reflex contractions (for example using triggering by tapping the skin of the suprapubic area).

There are further layers of complexity that will affect the precise urodynamic picture that a patient may exhibit. For example, bladder compliance is not determined simply by the level and completeness of the SCI but will be affected by the duration of time since injury and the way in which the urinary tract has been managed.[9]

Patients with lesions above the T6 levels and an intact distal autonomous cord are at risk of developing autonomic dysreflexia in response to noxious stimuli within the area innervated by the distal autonomous cord. Such stimulation produces a mass sympathetic nervous system response below the injury level which results in vaso-constriction below the injury level and a resultant rise in blood pressure. The hypertension seen with autonomic dysreflexia can be catastrophic. Removal of the noxious stimulus (e.g. by draining a distended bladder) will usually result in a rapid resolution of the crisis. Any clinician who is involved in the care of a patient with a SCI must be familiar with this condition and its management.

The clinical picture of the patient with an incomplete SCI is, of course, highly variable. Some patients will have an urodynamic picture that is indistinguishable from that seen in complete injuries except that a degree of preservation of sensation of bladder filling is present. On the other hand, others will have a preservation of an element of true voluntary bladder control. However, it is clear that urodynamic evaluation and follow up is needed in order not to underestimate the degree of lower urinary tract dysfunction.[10,11]

## AVAILABLE OPTIONS FOR PATIENT MANAGEMENT

Patients with SCI have a limited set of options available to them with regard to long-term urinary tract management. The choices that are made will depend on a number of different factors including personal acceptability, convenience, risks of short and long-term complications and the advice of medical staff, family and peers. It is interesting to note that different management approaches do not necessarily produce significant differences in general quality of life.[12]

It is immediately apparent that the natural pattern of lower urinary tract dysfunction that an individual with SCI may exhibit will preclude some forms of management unless urological interventions are used to alter lower urinary tract behavior. It is therefore the urologist’s role to widen the range of options open to patients. The available choices are:

- Clean intermittent (self) catheterization using the urethral route or a continent catheterizable abdominal conduit
- Contained urinary incontinence using a penile sheath system or pads
- Indwelling catheterization by the suprapubic or urethral route and using a catheter valve in some cases
- Voiding with an element of voluntary control which includes patient-induced or triggered reflex voiding, micturition by straining and, in some patients with incomplete injuries, true voluntary voiding.
- Sacral root stimulation using the Finetech-Brindley system in patients with complete supraconal injuries
- Urinary diversion by ileal conduit or continent urinary diversion

### Intermittent catheterization

Although there are infrequent circumstances where satisfactory arrangements can be made for intermittent catheterization to be performed by carers rather than the patient, in most situations such an arrangement is socially unsatisfactory. Therefore, intermittent catheterization is usually carried out by the patient themselves using a clean technique – clean intermittent self catheterization (CISC). The successful long-term use of the technique is dependent on patient motivation as the management system does involve a level of inconvenience. It is highly desirable that the patient should be reliably continent and have a bladder that stores an adequate volume of urine (400 ml or more) at low pressure.[13] The urologist may therefore have to treat neurogenic detrusor overactivity in a patient with a supraconal SCI or have to deal with reduced bladder compliance and/or stress incontinence in a patient with a conus injury. Options for treating detrusor overactivity include anticholinergic medication using the oral or intravesical route, intravesical botulinum toxin injections, augmentation cystoplasty or rhizotomy of the dorsal roots of S2, 3, 4.

Anticholinergic medication has a long-established and important role to play in improving bladder storage function after SCI.[14] Oral therapy is generally used as first line treatment for the patient who is incontinent while on CISC as a result of neurogenic detrusor overactivity. However, when oral treatment is not proving effective, a trial of intravesical oxybutynin can be undertaken as this may provide additional benefit in some cases.[15,16]

A level of enthusiasm has accompanied the introduction of intravesical botulinum injections into urological practice despite the somewhat limited evidence base from randomized controlled trials.[17] However, numerous case series have demonstrated the efficacy of the drug and reports are now emerging that suggest that a long-term program of regular re-injections is an acceptable alternative for some SCI patients who are keen to avoid major surgical procedures.[18]

When urine storage is compromised by reduced bladder compliance or intractable detrusor overactivity, augmentation cystoplasty has become established as a key tool in the urologist’s armamentarium.[19] The procedure is undoubtedly effective at producing a large-capacity, lowpressure storage reservoir but does represent a significant surgical intervention with associated immediate and long-term risks; careful patient counseling is vital prior to undertaking an ileo-cystoplasty.

The abolition of reflex detrusor contractions can also be successfully achieved by dividing the dorsal roots of S2, 3, 4. Although such a procedure has usually been accompanied by implantation of a Brindley sacral root stimulator (see below), it has been shown that a sacral bladder deafferentation can be successfully used in isolation in conjunction with CISC.[20] This report also emphasizes the fact that sacral dorsal rhizotomy can be a valuable tool in the treatment of autonomic dysreflexia when the condition is precipitated by a lower urinary tract or ano-rectal stimulus.

For patients who wish to use CISC but have problems with stress incontinence, a large number of surgical options are available while the value of medication is very limited. The choice of procedure is to some extent dependent on the preference of the surgeon as there is a paucity of evidence from clinical trials on which to base decision-making. For the SCI woman with stress incontinence, appropriate options would include the use of the rectus sheath fascial sling or the artificial urinary sphincter while in men the artificial sphincter has been widely used in such circumstances.[21] Where the urethra has been severely damaged, usually as a result of catheter-damage, urethral closure may be required; in women the technique described by Raz avoids the need for an abdominal incision.[22] In men, closure of the bulbar urethra rather than the bladder neck, has a similar benefit.

The use of a continent catheterizable abdominal conduit is a further development that is applicable to the SCI population. Although many different techniques for constructing such a conduit have been described, the Mitrofanoff principle is used most frequently;[23] the appendix is the conduit of choice but a short length of small intestine can be reconfigured into a narrow tube using the technique described by Monti if the appendix is not available.[24] Experience in both the SCI and wider populations have demonstrated that this is a viable reconstructive approach.[25] The construction of a catheterizable conduit can be undertaken in conjunction with other procedures such as an augmentation cystoplasty to create safe storage capacity or surgery to correct stress incontinence. Alternatively, it can be carried out in isolation if the lower urinary tract is capable of providing safe and reliable urine storage without additional surgical intervention.

The introduction of the continent catheterizable abdominal conduit into SCI urological practice has opened up the use of CISC to a wider population including the paraplegic who struggles with urethral self catheterization through limitations of mobility or problems with urethral sensitivity (in some incomplete SCI patients) and a small group of highly motivated and carefully selected quadriplegic patients.[26,27]

### Contained incontinence

Although many SCI patients will be naturally incontinent as a result of either reflex bladder contractions (neurogenic detrusor overactivity) or stress incontinence, their lower urinary tract will not necessarily be safely managed using either a penile sheath collection system or pads. It is vital that a urodynamic evaluation is carried out in order to gauge whether storage pressures within the bladder are likely to be safe and also to look for other relevant abnormalities such as the efficiency of bladder emptying or the presence of vesico-ureteric reflux. It is also important to ensure that the containment system that is used is effective and reliable so that the patient does not suffer from episodes where clothing or bedding gets wet.

Men with supraconal SCI have historically most frequently been managed using penile sheath collection. However, bladder emptying by neurogenic detrusor overactivity is often inefficient due to the effects of DSD and poorly sustained detrusor contractions with associated risks which include recurrent urinary tract infection and the development of hydronephrosis. In patients with injury levels above T6, symptoms of autonomic dysreflexia can be problematic if they arise during reflex bladder contractions. In order to address such issues, improved bladder emptying is required and can be achieved either through the use of additional CISC or by ablating the effect of the distal urethral sphincter.

The traditional approach to distal sphincter ablation for managing DSD has been to undertake an endoscopic division of the muscle – sphincterotomy. The surgical results of sphincterotomy are not completely reliable and repeat procedures may be needed although good long-term outcomes from endoscopic sphincterotomy have made this a standard procedure.[28–31] Alternative approaches to sphincter ablation have been explored in view of the fact that complications such as recurrent obstruction and impotence can arise after a standard sphincterotomy. Botulinum toxin injection into the external sphincter has been used by some authors but has not been widely adopted due to the technical difficulty of knowing if the injection has been accurately inserted and the need for regular re-injection.[32] Stents placed across the external urethral sphincter have also been used and have excellent short-term results but their use is limited by their expense and the complications that arise with long-term use – notably stent occlusion with tissue overgrowth or stones.[33–35]

Sphincterotomy can also be used in patients with conus injuries where emptying by either passive drainage or straining is inefficient as a result of residual sphincter tone (non-relaxing distal sphincter obstruction).

### Indwelling catheter drainage

For many years, a permanent indwelling urethral or suprapubic catheter was felt to represent suboptimal management for the patient with SCI. However, the role of such catheters has been re-evaluated and many spinal injuries services now regard the use of catheters as being a reasonable management option.[36–38] We should recall that the poor reputation of indwelling catheters was established during an era when catheter materials and manufacturing techniques were very different from those in use today.

For patients who opt for long-term catheterization, the convenience and reliability of catheter drainage are major factors in their decision-making. However, there are undoubtedly significant advantages to the use of suprapubic rather than a urethral catheter. Patients who lack sensation and/or have neurogenic detrusor overactivity are at high risk of developing urethral damage from pressure trauma to the urethra due to a catheter that is under tension, from catheter expulsions or as a result of peri-urethral sepsis. Suprapubic catheters also provide greater convenience for patients who are sexually active. Finally, there is limited evidence that infective complications may be seen less often in patients managed with suprapubic rather than urethral catheters.[39]

In recent years it has become apparent that many patients prefer to use a catheter valve, as opposed to continuous drainage into a bag. It is, of course, necessary to use urodynamic investigations to ensure that urine storage is occurring at safe pressures while the catheter valve is closed. Impaired storage as a result of detrusor overactivity, reduced compliance or stress incontinence can be treated in the same way as would be the case for the patient who is using CISC.

### Voiding with control

This approach is applicable to SCI patients who are able to achieve bladder emptying with an element of voluntary control and thereby achieve social continence. This group will include patients who have incomplete SCI, those who are able to efficiently trigger bladder emptying by inducing episodes of neurogenic detrusor overactivity and those who can strain to empty their bladders. However, it is critical that such patients are subject to careful evaluation and follow up as they are not immune from serious complications.[40]

### Sacral root stimulation

For patients with a complete supraconal SCI, the option of electrical stimulator-driven micturition can be considered. The Brindley system uses an external transmitter unit to induce an electrical signal in a subcutaneous receiver coil. The receiver is connected to electrodes that are placed around the ventral roots of S2, 3, 4. When activated, the system provides bursts of stimulation that contract both the detrusor smooth muscle and the striated urethral sphincter so that voiding does not occur as stimulation is applied. However, as soon as the stimulation is discontinued, voiding will occur because the striated sphincter relaxes immediately while the smooth muscle of the detrusor continues to contract. Bladder emptying is achieved by using a stimulation pattern consisting of bursts and gaps, with voiding occurring during the gaps. In most patients, the dorsal roots of S2, 3, 4 are divided in order to abolish reflex detrusor activity and promote continence.

Despite excellent results being reported from some centers, the Brindley system has had a somewhat limited impact on SCI urological management. This is a result of limited patient enthusiasm for a significant neurosurgical procedure and the associated effects of a dorsal rhizotomy (which include loss of reflex erection and ejaculation in men), the potential for side effects and complications and the cost of the equipment. However, the results from expert centers suggest that the device should remain in the armamentarium of the SCI unit.[41–43]

## UROLOGICAL COMPLICATIONS OF SCI

Despite careful patient evaluation and the introduction of an appropriate management program, the SCI patient remains at risk of developing urinary tract complications. In many circumstances, complications simply generate symptoms that are a nuisance to the patient but complications can also prove to be life-threatening. Long-term follow-up is therefore advised for the large majority of SCI patients; follow-up strategies are discussed below. The variety of different complications that are seen by the urologist with a SCI practice offers a continuous challenge to the clinical team. The following paragraphs describe some of the more important complications but do not provide a comprehensive list of all the problems that might be encountered.

### Stones

Both upper and lower urinary tract stones are commonly encountered. Upper tract stones are often infection-related so that, if neglected, renal loss can occur in association with the development of staghorn calculi.[44,45] The presenting symptomatology for upper tract stones in the SCI patient can be non-specific so that a high index of suspicion for stone disease is needed.[46] Early detection allows stone treatment when the stone burden is low and treatment can use conventional modalities – albeit with an added risk of side effects and complications.[7,47]

Bladder calculi are seen particularly commonly in patients on indwelling catheters and will often present with recurrent catheter blockages.[48] Such stones are not reliably detected by X-ray so that the patient whose catheter is blocking frequently is best assessed by means of cystoscopy.

### Urinary tract infection

UTI presents a formidable burden of ill-health to many SCI patients. For most, the problem simply amounts to recurrent bouts of symptoms while for others infection is associated with life-threatening sepsis or renal deterioration due to pyelonephritic scarring. Management strategies are based on the elimination of predisposing factors, general preventive measures and the appropriate use of antibiotics.[49]

Predisposing factors include persistent residual urine in patients using contained incontinence systems, stone disease and the use of catheterization (especially with indwelling catheters).[50] General preventive measures include maintenance of an adequate fluid throughput and the encouragement of regular bladder emptying (e.g. an adequate frequency of CISC or regular triggering of reflex bladder contractions).

Antibiotics remain a key factor in the control of management of UTI in the SCI population. However, inappropriate usage can lead to colonization of the patient with antibioticresistant organisms and exposes the patient to the side effect of antibiotics such as allergy and bowel disturbance. The development of, and adherence to, local antibiotic prescribing policies is to be encouraged and patient education is important if they are to accept an approach that limits inappropriate prescribing. Non-specialist clinicians also need to be educated in relation to the interpretation of urine culture results; asymptomatic bacteruria is almost inevitably present in patients with neuropathic lower urinary tracts so that a positive culture in isolation should not lead to antibiotics being prescribed.[51,52] Furthermore, the way in which samples are collected is important; the simple measure of changing an indwelling catheter before sample collection can reduce the inappropriate use of antibiotics.[53] The widespread use of low-dose antibiotic prophylaxis does not receive support from the majority of studies that have considered this approach.[54,55] However, very low levels of antibiotic administration in the prophylactic setting have been reported to reduce symptomatic UTI rates without increasing antibiotic resistance.[56]

New approaches to UTI management in SCI patients are undoubtedly needed. The use of bacterial interference by inducing colonization of the urinary tract with a nonpathogenic bacterial strain in order to prevent invasion by pathogenic strains has shown promise.[57]

### Renal failure

The high rates of renal failure that were seen in SCI patients in the decades immediately after the Second World War were the result of factors such as sepsis, hydronephrosis, renal calculi and amyloid disease.[58] Renal surveillance and improved lower urinary tract and general management have all contributed to greatly reduced rates of renal impairment in the SCI population. Despite the improvements, SCI patients continue to demonstrate an effect of their injury on renal integrity, especially as length of follow up increases.[59,60]

The relationship between renal deterioration and method of bladder management is of interest and both reflex voiding using penile sheath collection and the use of indwelling catheters have been associated with upper tract changes.[61,62] Pyelonephritic scarring in chronically catheterized patients may occur as a result of the bladder continuing to contract onto the catheter balloon with resultant impaired ureteric drainage;[63] the long-term significance of renal scarring in the SCI population is worthy of further study as it is of uncertain significance at present.[64]

### Bladder cancer

The risk of bladder cancer developing in a SCI patient has been the subject of significant interest although evidence now points towards the risk being similar to that of the able-bodied population.[65,66] However when tumors are seen they typically exhibit a squamous pattern and have a poor prognosis. Bladder surveillance is not justified on current evidence but hematuria or the development of new symptoms such as recurrent infections warrant urgent and thorough investigations.

## FOLLOW-UP PROTOCOLS

There is no doubt that SCI patients benefit from life-long follow-up and contact with the expertise offered by a Spinal Cord Injuries Unit. In the current era, such units are engaged in managing not only newly injured patients but also dealing with the effects of ageing on patients who have been on follow up for many years. Optimizing follow up regimes is important both for the individual patient and in order to utilize resources effectively. The intensity of urological follow-up is the subject of some debate.[67,68]

Guidelines from Europe have promoted relatively intensive follow up protocols while a UK group have proposed a simplified pattern of follow-up.[69,70] It is clear that followup should be tailored to the individual patient; for example, there is little value in undertaking repeated urodynamic studies on a patient who is managed using an indwelling catheter on free drainage.

There is general agreement that renal surveillance is appropriate in order to detect renal scarring, stones and hydronephrosis. Ultrasound is used extensively in this context with a policy of annual scanning in all patients representing a minimum level of follow-up.[70] Renography offers an opportunity to gather more detailed information regarding renal function and is therefore indicated in specific situations, such as when assessing a hydronephrosis that does not resolve with bladder drainage. However, more widespread use of renography in the screening context has also been suggested although the disadvantages of radiation exposure and cost limit the attraction of a screening role for renography.[71,72]

While urodynamic investigations will define the precise pathophysiological pattern of lower urinary tract dysfunction, there is less clarity as to whether an individual study will accurately predict the risk of a particular management strategy. Despite this, some authors promote the use of routine urodynamic studies in surveillance protocols;[73] others would take the view that this approach may lead to unnecessary interventions in a significant number of cases.[70]

The role of routine urine testing is also the subject of debate.[74] The use of dip test parameters to determine the need for formal culture has been suggested.[75]

## CONCLUSIONS

The management of the urinary tract of patients with SCI is continuing to evolve. Unfortunately, the scientific evidence base on which treatment decisions have to be made is poor. It is therefore important that clinicians take heed of the lessons that have been learnt in SCI centers over the decades but, at the same time, continue to question the accepted wisdom and subject it to scientific challenge.

The care of this group of patients is hugely rewarding for the treating clinical team as high-quality urinary tract management has a major positive impact on patients’ quality of life. On an international scale, there is an urgent need to devise cost-effective SCI management regimes that translate the results of the best SCI centers to the health care systems of the developing world.
